# Can erosions on MRI of the sacroiliac joints be reliably detected in patients with ankylosing spondylitis? - A cross-sectional study

**DOI:** 10.1186/ar3854

**Published:** 2012-05-24

**Authors:** Ulrich Weber, Susanne J Pedersen, Mikkel Østergaard, Kaspar Rufibach, Robert GW Lambert, Walter P Maksymowych

**Affiliations:** 1 Department of Medicine, Division of Rheumatology, 562 Heritage Medical Research Building, University of Alberta, Edmonton, Alberta, Canada T6G 2S2; 2 Department of Rheumatology, Balgrist University Hospital, Forchstrasse 340, CH-8008 Zürich, Switzerland; 3 Department of Rheumatology, Copenhagen University Hospital at Glostrup, Nordre Ringvej 57, DK-2600 Glostrup, Denmark; 4 Division of Biostatistics, Institute of Social and Preventive Medicine, University of Zürich, Hirschengraben 84, CH-8001 Zürich, Switzerland; 5 Department of Radiology and Diagnostic Imaging, University of Alberta, 2A2.41 Walter C. Mackenzie Health Sciences Centre, 8440-122 Street, Edmonton, Alberta, Canada T6G 2B7

## Abstract

**Introduction:**

Erosions of the sacroiliac joints (SIJ) on pelvic radiographs of patients with ankylosing spondylitis (AS) are an important feature of the modified New York classification criteria. However, radiographic SIJ erosions are often difficult to identify. Recent studies have shown that erosions can be detected also on magnetic resonance imaging (MRI) of the SIJ early in the disease course before they can be seen on radiography. The goals of this study were to assess the reproducibility of erosion and related features, namely, extended erosion (EE) and backfill (BF) of excavated erosion, in the SIJ using a standardized MRI methodology.

**Methods:**

Four readers independently assessed T1-weighted and short tau inversion recovery sequence (STIR) images of the SIJ from 30 AS patients and 30 controls (15 patients with non-specific back pain and 15 healthy volunteers) ≤45 years old. Erosions, EE, and BF were recorded according to standardized definitions. Reproducibility was assessed by percentage concordance among six possible reader pairs, kappa statistics (erosion as binary variable) and intraclass correlation coefficient (ICC) (erosion as sum score) for all readers jointly.

**Results:**

SIJ erosions were detected in all AS patients and six controls by ≥2 readers. The median number of SIJ quadrants affected by erosion recorded by four readers in 30 AS patients was 8.6 in the iliac and 2.1 in the sacral joint portion (*P *< 0.0001). For all 60 subjects and for all four readers, the kappa value for erosion was 0.72, 0.73 for EE, and 0.63 for BF. ICC for erosion was 0.79, 0.72 for EE, and 0.55 for BF, respectively. For comparison, the kappa and ICC values for bone marrow edema were 0.61 and 0.93, respectively.

**Conclusions:**

Erosions can be detected on MRI to a comparable degree of reliability as bone marrow edema despite the significant heterogeneity of their appearance on MRI.

## Introduction

Erosions of the sacroiliac joints (SIJ) on pelvic radiographs of patients with ankylosing spondylitis (AS) are an important feature of the modified New York classification criteria [1]. However, SIJ erosions are often difficult to identify on pelvic radiographs and training to recognize radiographic structural changes of the SIJ did not improve the performance of radiologists and rheumatologists in detecting radiographic sacroiliitis [2]. A comparison of SIJ radiographs with computed tomography (CT) scans showed a higher sensitivity of CT scans to detect structural changes indicative of sacroiliitis (86% versus 72%), but the same specificity (84%) [2]. However, assessment of SIJ erosions by CT in clinical practice is limited given two recent reports consistently indicating an association of malignancy with CT of the pelvis [3-5].

Recent studies have shown that erosions can also be detected on magnetic resonance imaging (MRI) of the SIJ early in the disease course before they can be seen on radiography [6] and that erosions may occur in the absence of bone marrow edema (BME) [7]. In the first report, 59% of non-radiographic spondyloarthritis (SpA) patients showed erosions on MRI in at least two SIJ quadrants [6]. The latter study demonstrated that recognition of erosions on T1-weighted spin echo (T1SE) MRI sequences contributes significantly to diagnostic utility in early SpA and that training to recognize lesions on T1SE MRI improves rheumatologist performance to diagnose SpA on MRI [7]. A recent retrospective analysis confirmed that erosion on SIJ MRI is a highly specific lesion in patients with SpA [8]. However, data on the reliability of detection of SIJ erosion by MRI are scarce [9,10].

**Figure 1:**
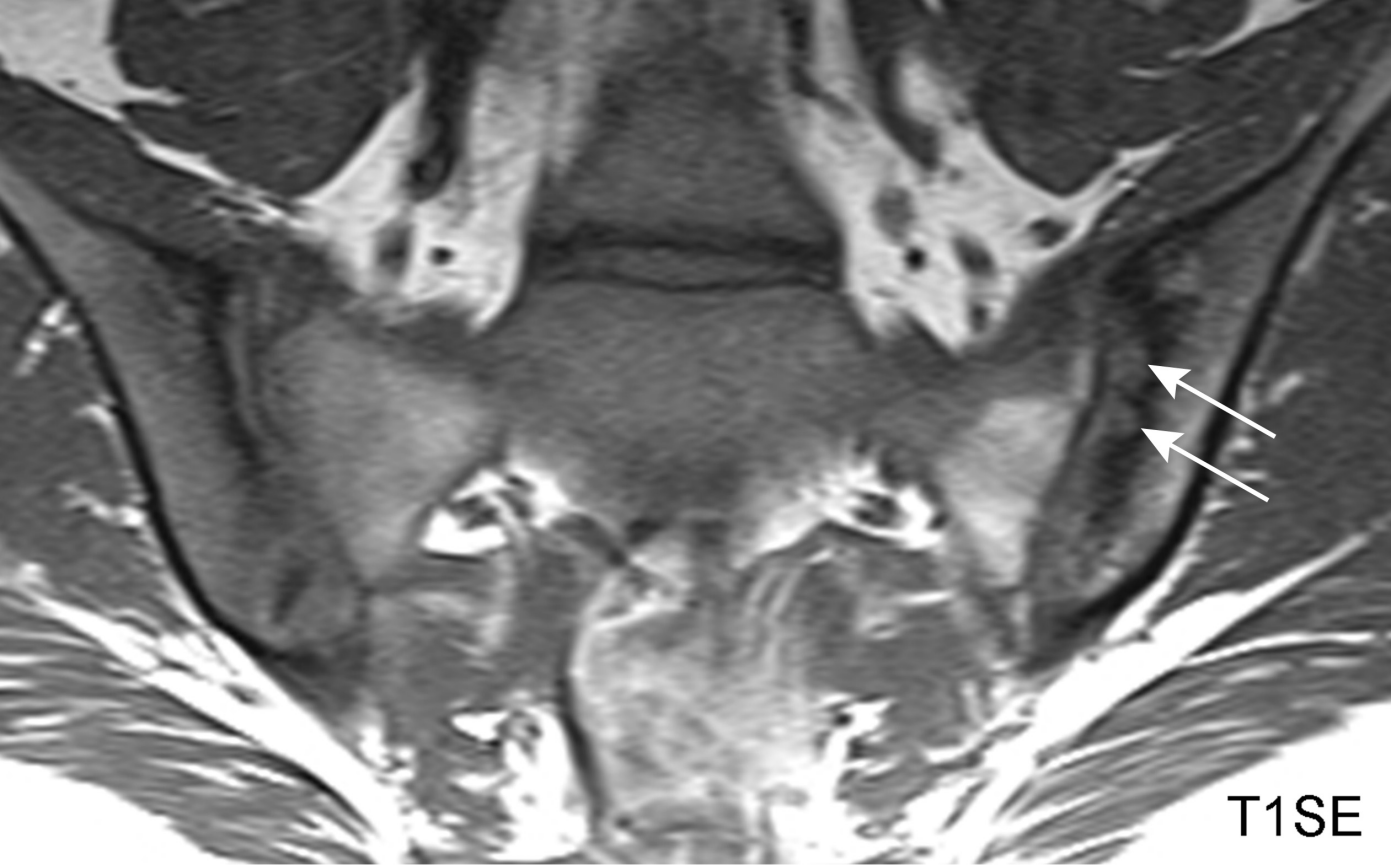
**Classical erosion (T1SE sequence)**. HLA B27 positive, 18-year-old female AS patient with inflammatory back pain for two years. Erosion in the left ilium (arrows) is characterized by loss of visualization of the cortical bone. The erosions are well visualized because they are surrounded by sclerosis as evidenced by very dark signal in the adjacent bone marrow. HLA-B27, human leucocyte antigen B27; T1SE, T1-weighted spin echo sequence.

Erosions may extend across major portions of the iliac and sacral subchondral bone and we call this feature extended erosion (EE). Previous descriptions of erosions on T1SE MRI have cited complete breech in subchondral bone with change of adjacent marrow signal as defining characteristics of an erosion (Figure 1) [6,7,11]. However, we have recently observed that the adjacent marrow signal may vary considerably and may even be increased on T1SE MRI suggesting tissue metaplasia. We have termed this novel appearance as 'backfill' (BF) because the appearance is consistent with reparative tissue re-filling an excavated erosion. The appearance of erosion on T1SE MRI may, therefore, vary considerably and it is essential to determine whether methodology can be sufficiently standardized and readers sufficiently calibrated to detect these lesions reliably.

## Materials and methods

### Subjects

The 60 subjects assessed in this study were randomly selected from a larger population of 187 SpA patients, non-specific back pain (NSBP) patients and healthy controls ≤45 years old, who were recruited in two rheumatology university hospitals [6]. The characteristics of the 30 AS patients meeting the modified New York criteria and of the 30 age- and sex-matched controls (15 NSBP patients and 15 healthy volunteers) are shown in Table 1. The median symptom duration in the two AS groups (15 patients each with symptom duration of ≤5 and > 5/≤10 years) was three and eight years, and 87% and 80% of the AS patients were HLA-B27 positive, respectively. Both AS groups had similar median Bath Ankylosing Spondylitis Disease Activity Index (BASDAI) [12] values close to 4 on a numeric rating scale from 0 to 10. AS patients with SIJ ankylosis or who received biologics within six months prior to the SIJ MRI were excluded. The local Ethics Committees approved the study protocol and written informed consent was obtained from all participants.

**Table 1:** Characteristics of the study participants and frequency of MRI lesions recorded by ≥2 readers.

Variable	AS ≤ 5 years duration (*n *= 15)	AS > 5/≤10 years duration (*n *= 15)	NSBP (*n *= 15)	HC (*n *= 15)
Male:female	8:7	9:6	10:5	8:7
Age, years	26.4 (2.8)	31.0 (9.5)	35.0 (8.5)	28.7 (4.8)
Symptom duration, years	3.0 (2.0)	8.0 (2.0)	N/A	N/A
HLA-B27 positive, %	86.7	80.0	N/A	N/A
BASDAI, NRS	4.2 (2.2)	3.9 (2.8)	N/A	N/A
BASFI, NRS	2.9 (4.2)	1.8 (2.0)	N/A	N/A
CRP level, mg/l	8.5 (12.3)	3.8 (2.5)	N/A	N/A
ER (%)	15 (100)	15 (100)	5 (33.3)	1 (6.7)
BME (%)	15 (100)	12 (80.0)	3 (20.0)	3 (20.0)
FI (%)	14 (93.3)	12 (80.0)	4 (26.7)	4 (26.7)
EE (%)	13 (86.7)	10 (66.7)	0 (0)	0 (0)
BF (%)	8 (53.3)	11 (73.3)	0 (0)	1 (6.7)

Values for study participant characteristics are the median (IQR) unless otherwise stated. AS, ankylosing spondylitis; BASDAI, Bath Ankylosing Spondylitis Disease Activity Index; BASFI, Bath Ankylosing Spondylitis Functional Index [29]; BF, backfill; BME, bone marrow edema; CRP, C-reactive protein; EE, extended erosion; ER, erosion; FI, fat infiltration; HC, healthy controls; HLA-B27, human leucocyte antigen B27; IQR, interquartile range; N/A, not applicable; NRS, numeric rating scale; NSBP, non-specific back pain.

### Reading exercise and MRI protocol

Four readers from three rheumatology university hospitals, who were blinded to the diagnosis and patient characteristics, independently assessed semicoronal T1SE and short tau inversion recovery (STIR) sequences of SIJ MRI scans in random order on electronic work stations. These are the sequences used in daily routine for MRI evaluation of SpA patients in the involved institutions. The detailed MRI parameters have been published previously [6,7]. The reading exercise of SIJ MRI of the 30 AS patients and the 30 non-specific back pain and healthy controls from the original cohort of 187 subjects was conducted two years after the original assessment of these MRI scans.

### Standardized assessment of MR images

**Figure 2:**
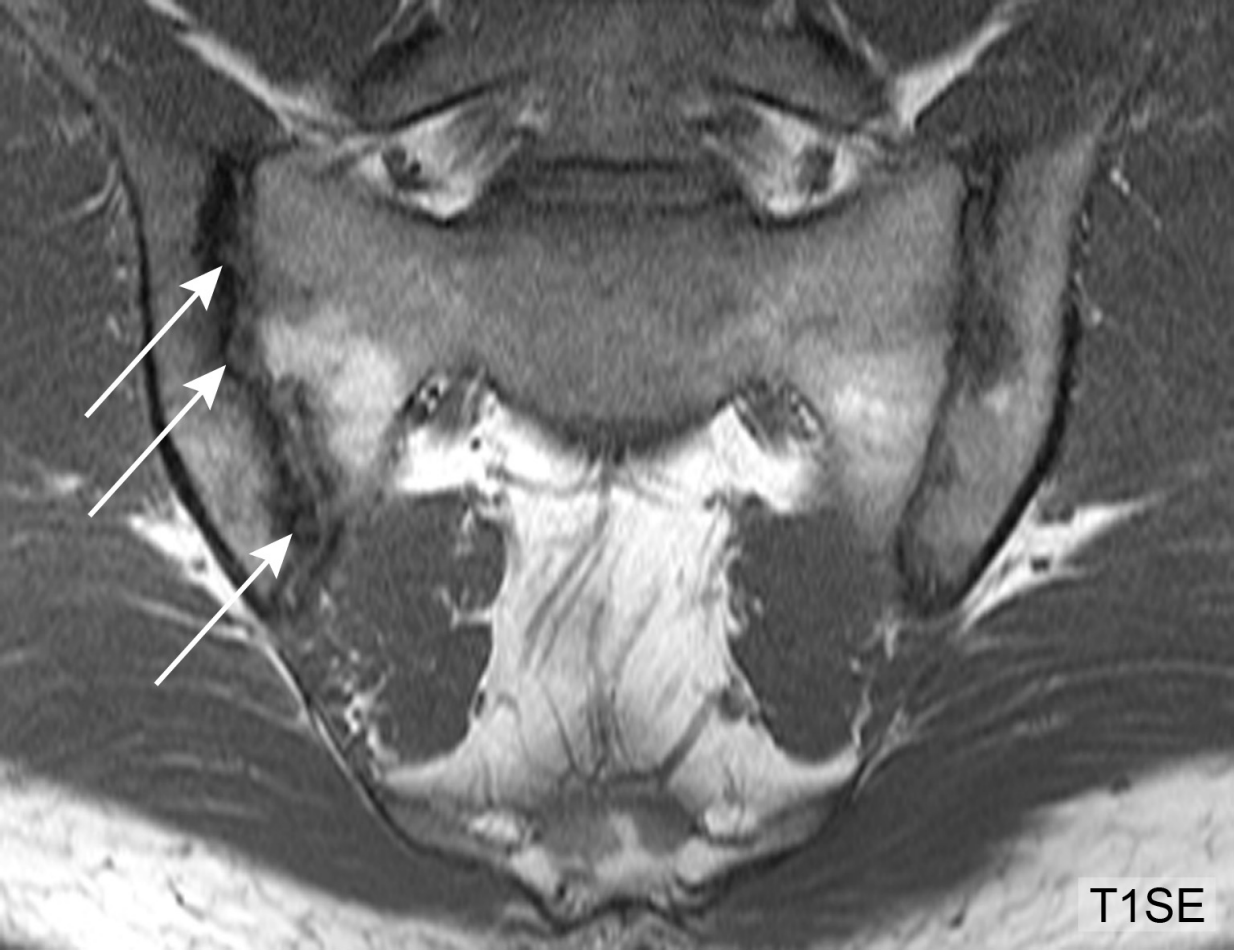
**Extended erosion (T1SE sequence)**. HLA B27 positive, 28-year-old male AS patient with inflammatory back pain for five years. The right ilium shows erosion which extends across the entire joint surface (arrows). HLA-B27, human leucocyte antigen B27; T1SE, T1-weighted spin echo sequence.

**Figure 3:**
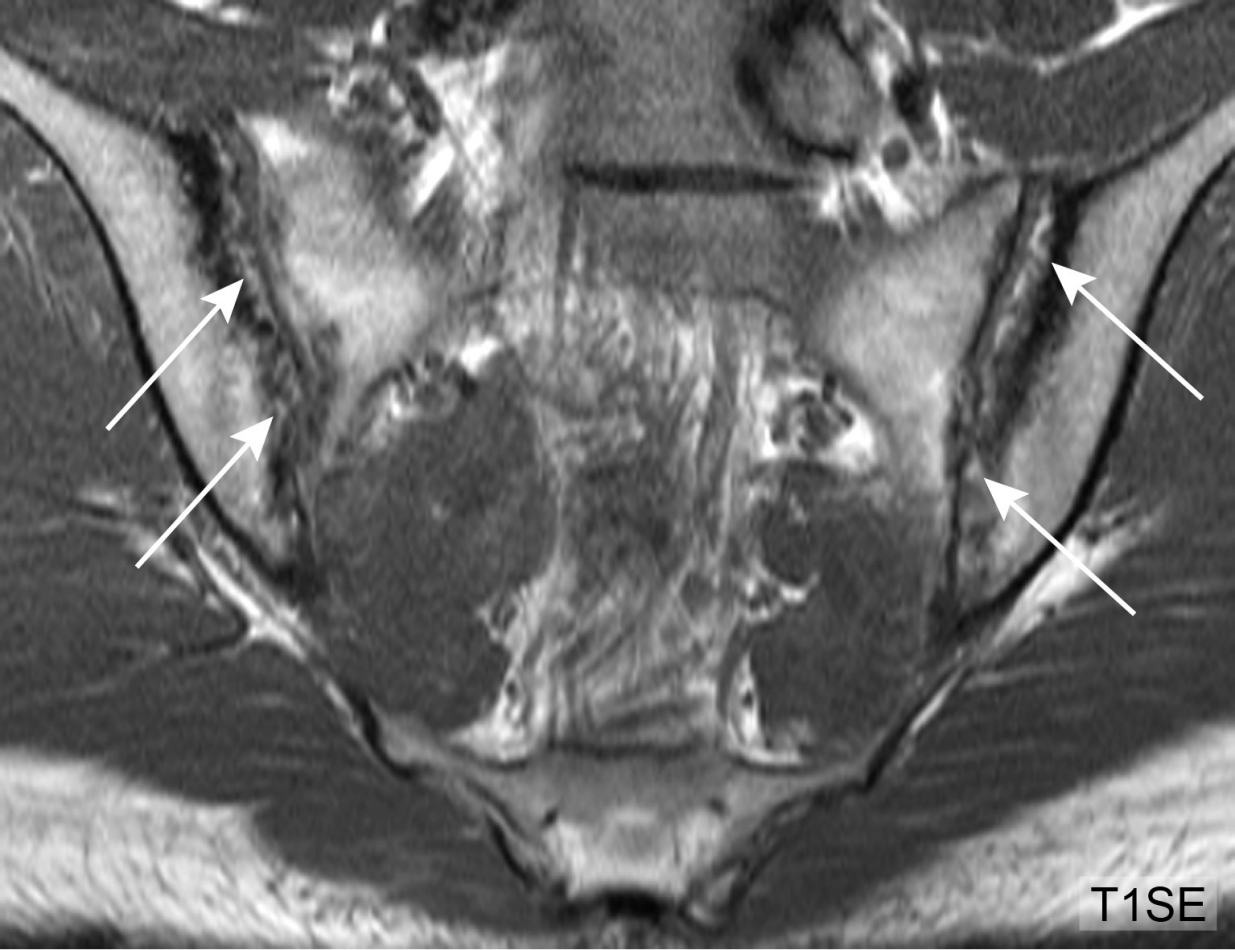
**Backfill, baseline MRI (T1SE sequence)**. HLA B27 positive, 25-year-old male AS patient. The T1SE sequence of the SIJ displays extended erosion of the iliac joint portion on both sides. The excavated iliac bone is replaced by tissue showing MR signal intensity similar to or higher than normal bone marrow (arrows). HLA-B27, human leucocyte antigen B27; MRI, magnetic resonance imaging; T1SE, T1-weighted spin echo sequence.

**Figure 4:**
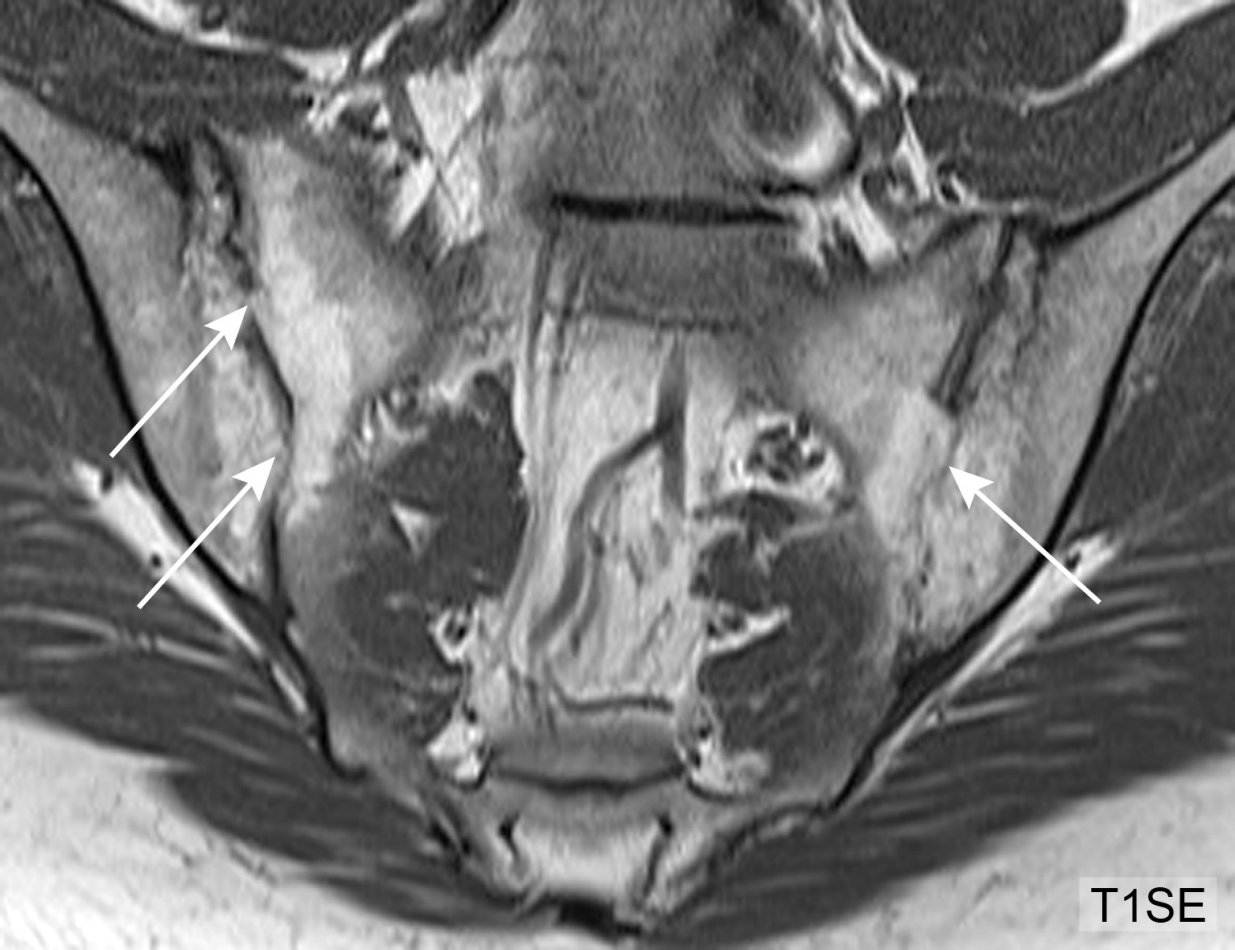
**Backfill, follow-up MRI after five years (T1SE sequence)**. Five years later, the same patient as in Figure 3 shows marked progression of structural lesions in both SIJ with ankylosis in previous areas of backfill (arrows). MRI, magnetic resonance imaging; SIJ, sacroiliac joint; T1SE, T1-weighted spin echo sequence.

We recorded lesions according to standardized definitions of active and structural lesions on SIJ MRI developed by the Canada-Denmark MR working group [13]. SIJ erosions are defined as full-thickness loss of dark appearance of either iliac or sacral cortical bone of the SIJ and change in normal bright appearance of adjacent bone marrow on T1SE images [6]. A retrospective analysis of the reading exercise in the original study population regarding diagnostic utility of SIJ MRI in SpA patients [6] focused on erosions and identified two main sources of inter-observer disagreement in reporting erosions. The first feature was extended erosion (EE). We standardized our approach by defining EE as erosion that extends continuously across the entire length of at least one SIJ quadrant of the iliac and/or sacral subchondral bone on the same semicoronal slice (Figure 2). The second feature was termed backfill (BF). BF is characterized by complete loss of iliac or sacral cortical bone with refilling of the excavated area by tissue which demonstrates comparable or even increased signal on T1SE sequence compared to reference normal marrow (Figures 3 and 4).

These newly described features representing the spectrum of abnormalities associated with SIJ erosions on T1SE MRI were added to a revised reference SIJ MR images set developed by consensus among study investigators [14]. This reference image set containing active and structural lesions served to calibrate the reader team. Calibration contained three international video-teleconference sessions using SIJ MRI scans of AS patients not involved in this study population. MR images of the SIJ were assessed according to the standardized methodology outlined in an online training module [13]. This standardizes the assessment of consecutive semicoronal slices through the SIJ from anterior to posterior outlining which are the first anterior and last posterior slices that are scored according to anatomical landmarks. We recorded erosions, bone marrow edema (BME) and fat infiltration (FI) in each SIJ quadrant on each semicoronal slice in a customized online data entry module described previously [6]. EE and BF were assessed per four iliac and sacral joint surfaces irrespective of the number of SIJ slices affected.

### Statistical analysis

#### Data description

At the patient level, we calculated frequencies of AS patients and controls with specific MRI abnormalities reported concordantly by ≥2 readers and also by all four readers. We also calculated the median (interquartile range (IQR)) number of quadrants showing erosion, BME and FI by ≥2 readers and also by all four readers. We repeated this analysis to assess frequencies of MRI lesions according to each of the four joint surfaces (right and left iliac, right and left sacral). We compared the frequencies of lesions observed in the iliac versus the sacral portions of the joint using the Wilcoxon test.

#### Interobserver reproducibility of specific MRI lesions

We calculated percentage agreement for specific MRI lesions according to positive and negative concordance among the six possible reader pairs for lesions detected at the four iliac and sacral joint surfaces per patient (total = 120 for each group). We also compared concordance separately for the iliac and sacral joint surfaces (total = 60 for each group).

Kappa statistics and intraclass correlation coefficients (ICC) were used to calculate the reproducibility of specific MRI lesions for all four readers jointly and for all 60 study participants (30 AS patients and 30 controls), per iliac and sacral joint portion, and per subject. The inter-reader agreement was defined as slight, fair, moderate, substantial and almost perfect by values of the estimated Cohen's kappa κ < 0.2, 0.2 ≤κ < 0.4, 0.4 ≤κ < 0.6, 0.6 ≤κ < 0.8, and 0.8 ≤κ < 1, respectively [15]. Among the six reported variants of ICC, we report the results of the ICC(3, 1) and ICC(2, 1) model [16-18]. The ICC(3, 1) approach considers the study readers to be a fixed sample and thus not representative of a larger population of raters. In the ICC (2, 1) approach, study readers are considered to be a random sample and, therefore, representative of a larger population of raters. ICC values > 0.4, > 0.6, > 0.8, and > 0.9 were regarded as representing moderate, good, very good, and excellent reproducibility, respectively. For kappa, we provide bootstrap confidence intervals based on 1,000 bootstrap replications. All confidence intervals are computed using a confidence level of 95% and statistical tests are considered significant if the *P*-value is ≤0.05.

## Results

### Data description

#### Frequency of MRI lesions in the AS and control groups

**Figure 5:**
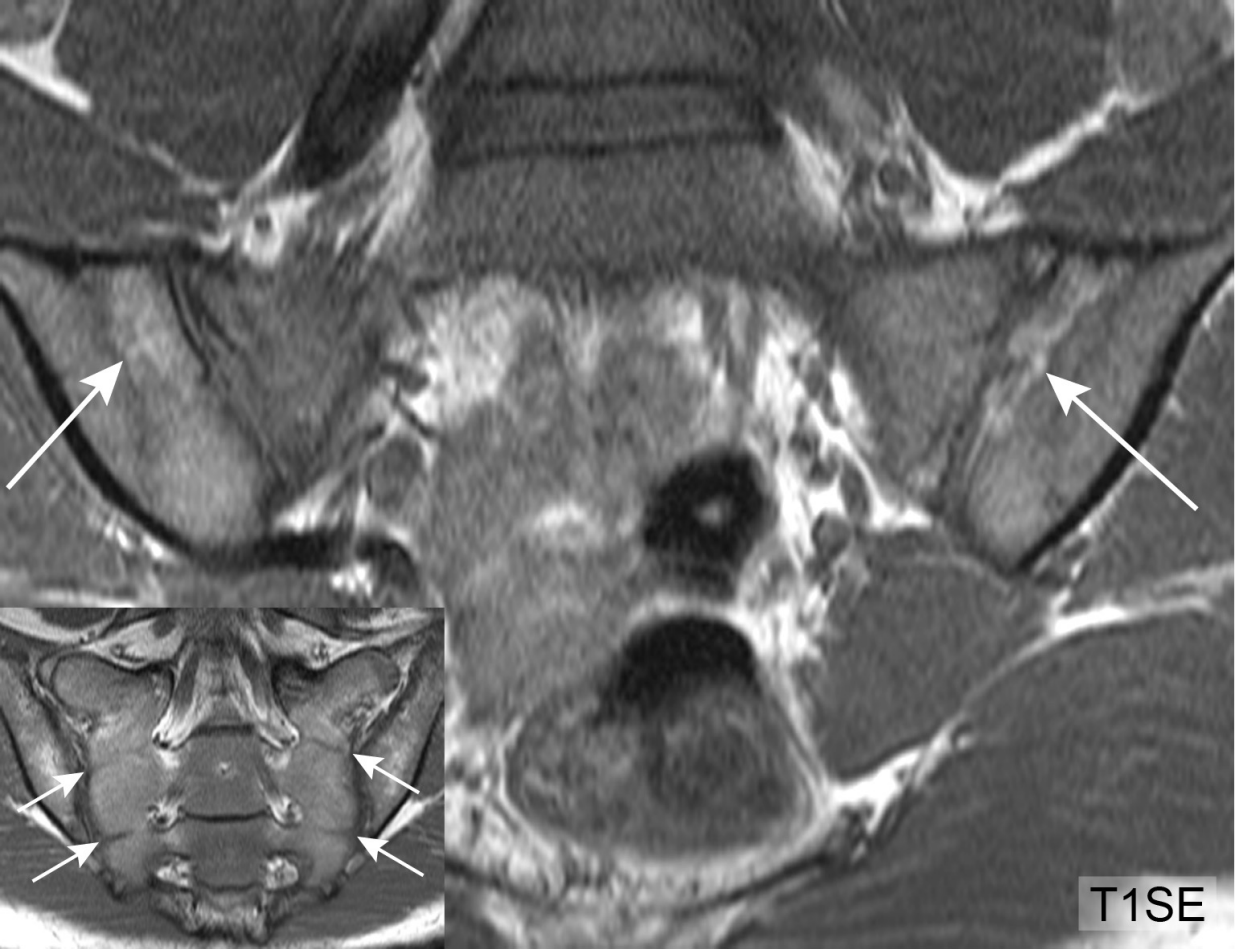
**Open epiphyseal growth plate mimicking backfill (T1SE sequence)**. 18-year-old male healthy control. Irregularly shaped increased signal alteration on T1SE sequence (arrows) compatible with fat infiltration of the anterior predominantly iliac joint portion. Insert: The sacral vertebrae are not yet completely fused (arrows). T1SE, T1-weighted spin echo sequence.

Erosions on SIJ MRI were detected in all 30 AS patients by ≥2 readers, whereas five NSBP patients and one healthy control (20% of all controls) also showed lesions meeting the definition of erosion (Table 1). EE and BF were recorded in 23 (76.7%) and 19 (63.3%) of the AS patients, respectively. Fifteen (78.9%) of the 19 AS patients showing BF also displayed EE. No EE was observed in the 30 controls, but an MRI lesion meeting the definition of BF was reported by two readers on SIJ MRI of a healthy control. At post-hoc debriefing of all readers the consensus was that the increased signal alteration on T1SE in this control represented a still open epiphyseal growth plate in an 18-year-old man (Figure 5). BME was detected by ≥2 readers in 27 (90%) of the AS patients and in six (20%) controls, whereas FI was recorded in 26 (86.7%) and eight (26.7%) of the AS patients and controls, respectively.

The median number of SIJ quadrants showing erosions as recorded by four readers in 30 AS patients was statistically significantly higher in the ilium (8.6, IQR 6.9) than in the sacrum (2.1, IQR 2.9; *P *< 0.0001) (Table 2). EE and BF were also observed statistically significantly more frequently in the iliac joint part (*P*-value for both lesions < 0.0001). BME and FI showed no statistical difference in their distribution between the ilium and sacrum. On a patient level, BME was the most frequently reported MRI lesion in the AS group (median 13.1 SIJ quadrants, IQR 15.1). The frequency of erosions, BME and FI per subject was too low in the 30 controls to draw conclusions about a preferential distribution of these features in the ilium or sacrum. The MRI feature observed most frequently in the control group was FI.

**Table 2:** Frequency of MRI lesions recorded by four readers jointly and percentage concordance among six possible reader pairs.

	Lesion	Per subject	Ilium R+L	Sacrum R+L	*P*-value Ilium versus Sacrum
AS patients (number = 30)					
Frequency: median (IQR)^a^	ER	11.4 (9.0)	8.6 (6.9)	2.1 (2.9)	< 0.0001
	BME	13.1 (15.1)	6.8 (10.4)	5.1 (7.9)	0.67
	FI	10.9 (11.7)	4.5 (5.3)	5.6 (7.0)	0.06
	EE	1.1 (1.4)	1.0 (1.0)	0.0 (0.3)	< 0.0001
	BF	1.0 (1.3)	0.8 (1.3)	0.0 (0.3)	< 0.0001
% Concordance six RP: total (positive/negative)^b^	ER	73.9 (56.9/16.9)	80.0 (75.0/5.0)	67.8 (38.9/28.9)	N/A
	BME	85.0 (60.4/24.6)	79.7 (60.3/19.4)	90.3 (60.6/29.7)	N/A
	FI	69.7 (43.6/26.1)	66.4 (35.3/31.1)	73.1 (51.9/21.1)	N/A
	EE	75.7 (19.7/56.0)	65.8 (32.5/33.3)	85.6 (6.9/78.6)	N/A
	BF	80.0 (13.8/66.3)	70.3 (24.7/45.6)	89.7 (2.8/86.9)	N/A
Controls (number = 30)					
Frequency: median (IQR)^a^	ER	0 (0.4)	0 (0.3)	0 (0.0)	0.02
	BME	0.1 (0.9)	0 (0.4)	0 (0.3)	0.81
	FI	0 (2.1)	0 (0.9)	0 (0.2)	0.26
	EE	0 (0.0)	0 (0.0)	0 (0.0)	N/A
	BF	0 (0.0)	0 (0.0)	0 (0.0)	0.98
% Concordance six RP: total (positive/negative)^b^	ER	92.1 (0.8/91.3)	87.8 (1.4/86.4)	96.4 (0.3/96.1)	N/A
	BME	89.3 (1.9/87.4)	88.6 (2.2/86.4)	90.0 (1.7/88.3)	N/A
	FI	83.8 (2.9/80.8)	80.3 (4.7/75.6)	87.2 (1.1/86.1)	N/A
	EE	100.0 (0.0/100.0)	100.0 (0.0/100.0)	100.0 (0.0/100.0)	N/A
	BF	98.7 (0.2/98.5)	98.1 (0.3/97.8)	99.2 (0.0/99.2)	N/A

^a^Frequency of lesions: for ER, BME and FI the number of SIJ quadrants affected; for EE and BF the number of joint surfaces affected irrespective of the number of SIJ MRI slices; ^b^percentage concordance among six possible reader pairs for 60 joint surfaces (Ilium R+L; Sacrum R+L) and for 120 joint surfaces (per subject). AS, ankylosing spondylitis; BF, backfill; BME, bone marrow edema; EE, extended erosion; ER, erosion; FI, fat infiltration; IQR, interquartile range; L, left; N/A, not applicable; R, right; RP, reader pair; % Concordance six RP, percentage concordance among six possible reader pairs (100% = six concordant reader pairs); total = positive and negative concordance.

### Reproducibility of MRI lesions

#### Percentage concordance among six possible reader pairs

Among the 30 AS patients, the percentage concordance for the detection of erosions in the iliac joint portion was high (80.0%; positive/negative concordance - 75.0% and 5.0%, respectively) and comparable with the percentage concordance for BME (79.7%; positive/negative concordance - 60.3% and 19.4%, respectively) (Table 2). The percentage concordance in the sacral joint portion, which had a statistically significantly lower frequency of erosions compared with the ilium, showed a lower concordance between the six possible reader pairs for erosion (67.8%) than for BME (90.3%) resulting in a lower percentage concordance also at the patient level (73.9% for erosion versus 85.0% for BME). The lowest percentage concordance was observed for FI, both for the AS and the control group (69.7% and 83.8%, respectively, per patient level). At the patient level, the percentage concordance for EE (75.7%) and BF (80.0%) was similar to erosion (73.9%). The percentage concordance for the control group, where comparatively few lesions were observed, was high both for erosion (92.1%) and BME (89.3%).

#### Kappa statistics (MRI lesions as binary variables) for four readers jointly

For the 60 study participants, reader agreement expressed by kappa values was substantial and comparable for erosion and for BME at the subject level (for erosion 0.72, 95% confidence interval (CI) 0.57 to 0.84, and for BME 0.61, 95% CI 0.47 to 0.74, respectively) and for the ilium (for erosion 0.67, 95% CI 0.53 to 0.79, and for BME 0.64, 95% CI 0.50 to 0.76, respectively) (Table 3). Kappa values were significantly lower in the sacrum for erosion (0.56, 95% CI 0.42 to 0.70) than for BME (0.71, 95% CI 0.57 to 0.82), but the frequency of erosion was also significantly lower in the sacral portion. At the subject level, the kappa values for EE (0.73, 95% CI 0.63 to 0.84) and for BF (0.63, 95% CI 0.53 to 0.73) indicated substantial agreement comparable to the kappa values of erosion and BME. Despite a lesion frequency similar to erosion, FI showed the lowest kappa value (0.55, 95% CI 0.41 to 0.68).

**Table 3:** Agreement by kappa statistics and intraclass correlation coefficients by four readers jointly and for 60 study subjects.

	Lesion	Per subject	Ilium R+L	Sacrum R+L
Kappa value (95% CI)	ER	0.72 (0.57 to 0.84)	0.67 (0.53 to 0.79)	0.56 (0.42 to 0.70)
	BME	0.61 (0.47 to 0.74)	0.64 (0.50 to 0.76)	0.71 (0.57 to 0.82)
	FI	0.55 (0.41 to 0.68)	0.45 (0.32 to 0.58)	0.60 (0.46 to 0.72)
	EE^a^	0.73 (0.63 to 0.84)	0.68 (0.58 to 0.79)	0.45 (0.35 to 0.56)
	BF^a^	0.63 (0.53 to 0.73)	0.60 (0.50 to 0.70)	0.32 (0.22 to 0.42)
ICC(3, 1)/ICC(2, 1)	ER	0.79/0.75	0.78/0.74	0.71/0.67
	BME	0.93/0.92	0.88/0.86	0.94/0.93
	FI	0.71/0.63	0.54/0.46	0.75/0.70
	EE^a^	0.72/0.71	0.65/0.63	0.55/0.55
	BF^a^	0.55/0.55	0.59/0.59	0.35/0.35

^a^EE and BF were assessed as score per four joint surfaces irrespective of the number of SIJ MRI slices affected. Kappa values: MRI lesion as binary variable per subject. Intraclass correlation coefficients: MRI lesion as sum score per subject. AS, ankylosing spondylitis; BF, backfill; BME, bone marrow edema; CI, confidence interval; EE, extended erosion; ER, erosion; FI, fat infiltration; ICC(3, 1), intraclass correlation coefficient (3, 1): Readers are considered not representative of a larger population of readers; ICC(2, 1), Intraclass correlation coefficient (2, 1): Readers are considered representative of a larger population of readers.

#### Intraclass correlation coefficients (MRI lesions as sum scores) for four readers jointly

At the subject level, reader agreement regarding sum scores of the 60 study participants was higher for BME (ICC(3, 1) 0.93 and ICC(2, 1) 0.92, respectively) than for erosion (ICC(3, 1) 0.79 and ICC(2, 1) 0.75, respectively) (Table 3). This difference was also observed for both the iliac and sacral joint portion. Consistent with reader pair percentage concordance and kappa values, assessment of FI also had the lowest reliability according to ICC values (ICC(3, 1) 0.71 and ICC(2, 1) 0.63, respectively). Assessment of EE and BF was less reliable than of erosions overall but assessment for these two lesions was based on evaluation of the four cortical surfaces in the entire joint as compared to the erosion score based on evaluation of eight SIJ quadrants.

## Discussion

This systematic and controlled evaluation of erosion and related features on SIJ MRI in patients with AS had two findings of clinical relevance. First, the reliability of detecting erosion on SIJ MRI was substantial and comparable to BME, provided that readers are trained to recognize abnormalities on T1SE MRI. Familiarity with the variable features of erosions on MRI may also improve reliability. These features include EE and BF. Second, erosions occurred statistically significantly more frequently in the ilium compared with the sacrum. This finding has to be taken into account when comparing various lesions detected on SIJ MRI in AS patients, because BME and FI showed a similar distribution across both joint surfaces.

A debriefing analysis of the reading exercise in the original study population [6] retrospectively identified EE and BF as major sources of inter-observer disagreement regarding erosion. It is possible that these appearances represent stages in the evolution of an erosive lesion because 15 (78.9%) of 19 AS patients showing BF also showed EE. We, therefore, consider it appropriate to incorporate these features into a comprehensive definition of SIJ erosion. We interpret the signal alteration of BF on T1SE sequences as metaplastic tissue which refills the excavated subchondral bone. For obvious ethical reasons, histopathologic evidence in support of this assumption is not available. Serial MRI assessments of the SIJ over several years may help determine if BF represents an intermediate stage between SIJ erosion and ankylosis. In contrast to rheumatoid arthritis, where regression of erosion has been reported only rarely [19,20], erosion in many SpA patients may be followed by refilling of the excavated bone and eventually ankylosis.

Two previous reports focused on agreement data for structural MRI lesions of the SIJ [9,10]. In a cohort of 68 patients with inflammatory back pain according to Calin criteria, the concordance rate for structural lesions defined as a composite index of ankylosis, sclerosis and erosions was 81% and 88%, and kappa values were 0.37 and 0.66, respectively [9]. However, differences in study design preclude a direct comparison with our reliability data. Unlike our study cohort, the study population consisted of patients with inflammatory back pain and only 14 patients (20.6%) met the radiographic modified New York criteria, structural MRI lesions were less frequent (16%), assessment was based on different definitions of MRI lesions, lesions were recorded for the entire right and left SIJ rather than according to joint surface, and there was no control group. Another study which evaluated SIJ MRI in 41 inflammatory back patients meeting the European Spondylarthropathy Study Group (ESSG) criteria [21], reported an interreader agreement (percentage agreement/kappa value) between two senior radiologists of 77%/0.54 for erosion [10]. The lesion analysis according to eight SIJ quadrants noted a predominance of erosions in the iliac joint portion, but no formal comparison of lesion frequency between the two joint surfaces was performed. Again, there were major differences in study design compared to our work regarding study population, MRI lesion definitions and imaging technique, and lack of a control group.

Our study was conducted with T1SE and STIR sequences representing the routine protocol used for MRI evaluation of SpA patients. Additional so-called 'cartilage MRI sequences', such as T1-weighted fat saturated (T1FS) or T2-weighted gradient echo (T2GE) sequences, may offer advantages to recognize erosion. However, they require further evaluation as to their reliability for detection of erosion compared to T1SE sequence alone and their implementation into routine MRI scanning protocols with regard to examination time and costs [22]. A study using both T1SE and T1FS sequences to detect SIJ erosions in 37 SpA patients meeting the ESSG criteria reported a good inter-observer agreement by kappa statistics between two trained radiologists (0.76 for erosion at joint level and 0.80 at patient level) [10]. Erosion was defined as loss of marrow signal on T1SE and T1FS images together with a defect in the overlaying cortical bone; erosions were scored regarding their extent on the joint surface and the presence of ankylosis was added to the erosion score. However, this study did not directly compare T1SE and T1FS sequences with regard to reliability for detection of SIJ erosions, a separate analysis without the contribution of ankylosis to the erosion score was not performed, and there was no control group. A recent report compared T1FS sequences with two variants of T2GE sequences, three-dimensional -fast low angle shot (3D-FLASH) and three-dimensional-double excitation in the steady state (3D-DESS) sequences, in a retrospective analysis of scans of 30 patients with clinically suspected sacroiliitis and nine healthy controls [23]. There was no difference in the number of erosions detected by all three sequences, but erosion scores based on the extent of joint involvement were significantly higher in both T2GE sequences compared with T1FS sequences. The reliability for detection of erosion by these three 'cartilage MRI sequences' could not be assessed because only one reader evaluated the scans and there was no comparison with the T1SE sequence often used in daily routine.

There are no controlled data on whether CT may have higher sensitivity to detect SIJ erosions compared with MRI. However, two recent studies consistently reported an increased risk of malignancy associated with CT of the pelvis [3-5]. The adjusted lifetime attributable risk of cancer was two cancers per 1,000 exposed women for 20-year-old women who undergo pelvic CT examination [4]. This concern about radiation dose may limit the use of CT to assess SIJ erosions in daily routine and particularly in studies involving healthy controls.

We found a marked difference for erosion kappa values in the iliac versus the sacral joint portion. However, kappa values depend on the prevalence of the finding under observation [24-26]. Kappa values for erosion and BME were virtually identical in the ilium, which may be partly due to a higher occurrence of erosion in this area, comparable to the occurrence of BME. In contrast, erosion kappa values for the sacrum were lower than for BME which occurred more frequently than erosion in the sacrum. Another reason for the difference in detecting erosion between the ilium and sacrum may be different MRI appearances of erosion in the two articular surfaces. Such differences in erosion phenotype may relate to cartilage thickness (which is usually thinner on the iliac compartment), size and depth of erosions, or MRI artifacts, such as chemical shift, which may impair the assessment of subchondral bone [27,28].

## Conclusions

This systematic, standardized, and controlled evaluation of SIJ MRI scans in AS patients demonstrated that the reliability between four readers for detection of erosion on SIJ MRI was substantial and comparable to BME and that, in contrast to BME and FI, erosion occurred significantly more frequently on the iliac side. The spectrum of appearance of erosion on MRI is much more heterogeneous than reported previously and recognition of variants such as 'extended erosion' and 'backfill' may facilitate overall detection of erosion. Moreover, further assessment in prospective studies is required to understand the characteristics of these variants and their role in the evolution of sacroiliitis.

## Abbreviations

AS: ankylosing spondylitis; BASDAI: Bath Ankylosing Spondylitis Disease Activity Index; BASFI: Bath Ankylosing Spondylitis Functional Index [29]; BF: backfill; BME: bone marrow edema; CI: confidence interval; CRP: C-reactive protein; CT: computed tomography; EE: extended erosion; ER: erosion; ESSG: European Spondylarthopathy Study Group; FI: fat infiltration; ICC: intraclass correlation coefficient; IQR: interquartile range; HC: healthy control; HLA-B27: human leucocyte antigen B27; MRI: magnetic resonance imaging; N/A: not applicable; NRS: numeric rating scale; NSBP: non-specific back pain; RP: reader pair; SIJ: sacroiliac joint; SpA: spondyloarthritis; STIR: short tau inversion recovery sequence; T1FS: T1-weighted fat saturated sequence; T1SE: T1-weighted spin echo sequence; T2GE: T2-weighted gradient echo sequence.

## Competing interests

The authors declare that they have no competing interests.

## Authors' contributions

MØ, RL, SP, UW and WM drafted the study design. UW and WM acquired the clinical data. MØ, SP, UW and WM were MRI readers. KR and UW performed the statistical analysis. All authors take responsibility for interpretation of data. UW drafted the manuscript with contributions from all authors. All authors read and approved the final manuscript.
